# Mitochondrial genome of captive Alpine musk deer, *Moschus chrysogaster* (Moschidae), and phylogenetic analyses with its coordinal species

**DOI:** 10.1080/23802359.2021.1875930

**Published:** 2021-02-13

**Authors:** Chao Yang, Wei-Feng Wang, Xiao-Juan Du, Xiao Tan, Li-Juan Suo, Kun Bian, Fei-Ran Li, Jie Tang, Ben-Mo Jiang, Xue-Juan Li, Yan Wang

**Affiliations:** a Shaanxi Institute of Zoology, Xi’an, PR China; b Shaanxi Nature Reserve and Wildlife Management Station, Xi’an, PR China; c Sichuan Fengchun Pharmaceutical Co. Ltd, Chengdu, PR China; d School of Life Sciences, Shaanxi Normal University, Xi’an, PR China

**Keywords:** *Moschus chrysogaster*, high-throughput, mitogenome, phylogeny

## Abstract

Alpine musk deer, *Moschus chrysogaster*, a solitary, primitive ungulate inhabiting high elevation areas (3000–4500 m) is an endangered species facing threat of extinction globally due to excessive hunting for its musk. In this study, we determined the complete mitochondrial genome of *M. chrysogaster*, which was 16,354 bp in length, and revealed the same gene order and genomic organization as typical Moschidae mitochondrial DNA. Start codons in 13 protein-coding genes (PCGs) were all typical ATGs except ATA for *ND2* and *ND3* and ATT for *ND5*. Stop codons were all typical types except an incomplete stop codon T for *COX3*, *ND2*, *ND3*, and *ND4*. Secondary structures in 22 transfer RNA genes all showed typical cloverleaf except *tRNA-Ser* (AGY), in which the dihydrouridine arm formed a simple loop. No repeat units were found in the control region. The topology structure indicated that *M. cupreus* was primitive and located at the root of the Moschidae clade. Phylogenetic reconstruction placed *M. chrysogaster* as a distinct lineage, closely related to the branch of *M. leucogaster*, *M. berezovskii* (wild) and predicted a sister relationship with *M. moschiferus*, *M. anhuiensis,* and *M. berezovskii* (captive). However, we suggested that the genetic resources of *M. chrysogaster_*JQ608470 should be further investigated.

As an endangered species, Alpine musk deer, *Moschus chrysogaster*, was listed on the IUCN Red List (Harris 2016) and categorized as a first-degree national protected species in China. This species occurs from the highlands of central China (the Helan Mountains from the northern edge of its distribution), to areas south and west of the Himalayas, extending to eastern Nepal, Bhutan, and northeastern India (Wemmer 1998). It is widely but discontinuously distributed across the mountainous parts of the Himalayas (Aryal et al. 2010). Owing to excessive hunting, as well as habitat loss and degradation, wild populations have been declining for decades (Meng et al. 2003). Only one legal institution exists in China to breed *M. chrysogaster* in captivity, and efforts are also under way to do so in India (Mithileshwari et al. 2016). To domesticate, genetically improve, and systematically conserve wild populations, it is necessary to define the genetic characteristics of *M. chrysogaster* (Yang et al. 2013).

The muscle tissue used for DNA extraction and analysis was sampled from a captive male Alpine musk deer, *M. chrysogaster*, that died of natural causes in Feng County, Shaanxi Province (N: 34.231295, E: 106.903539), and the specimen (voucher number: MS01) was deposited in the animal specimen museum of the Shaanxi Institute of Zoology, Xi’an, China (contacts: Chao Yang, chaoy819@xab.ac.cn).

Total genomic DNA was extracted to construct paired-end libraries, tagged, and subjected to the high-throughput Illumina Xten platform sequencing with a 150 bp paired-end strategy. Clean reads were trimmed by removing regions with a Phred score of <10 and 7,259,638 paired-end raw reads were obtained. Then, MITObim version 1.9 (Hahn et al. 2013) was used to assemble the clean reads with the complete mitochondrial genome (mitogenome) of *M. berezovskii* (GenBank: MH047347) as a reference. A total of 79,140 individual mitochondrial reads were mapped to the reference mitogenome, giving an average coverage of 709.8X. Geneious version 2020.0.4 (Kearse et al. 2012) and the MITOchondrial genome annotation server (MITOS; http://mitos.bioinf.uni-leipzig.de/index.py) were used for annotation of protein-coding genes (PCGs) in the mitogenome, and data were manually inspected to predict transfer RNA (tRNA) and ribosomal RNA (rRNA) genes.

Finally, the whole mitogenome sequence of *M. chrysogaster* was obtained and submitted to GenBank (accession number MW284875); the genome was 16,354 base pairs (bp) in length, including 13 PCGs, 2 rRNA genes, 22 tRNA genes, and one control region. Gene content and arrangement were identical to those of other typical mammalian mitogenomes (Pan et al. 2015). The base composition of the *M. chrysogaster* mitogenome was as follows: A = 34%, T = 28%, C = 25.1%, G = 12.9%, and A + T = 62%. With the exception of *ND2* and *ND3*, which start with ATA, and *ND5*, which starts with ATT, all PCGs had typical ATG start codons, and all of them ended with a complete triplet codon (TAA and AGA), except for *COX3*, *ND2, ND3*, and *ND4*, which ended with an incomplete T.

The length of 12S rRNA was 956 bp, and that of 16S rRNA was 1569 bp, located between *tRNA-Phe* and *tRNA-Leu*(UUR), separated by *tRNA-Val*. The length of the 22 tRNA genes ranged from 60 bp (*tRNA-Ser*(AGY) to 75 bp (*tRNA-Leu*(UUR)), and all tRNA genes had typical cloverleaf secondary structures, with the exception of *tRNA-Ser*(AGY), in which the dihydrouridine arm formed a simple loop. The control region was 924 bp in length, located between *tRNA-Pro* and *tRNA-Phe*, and the AT content was 63.6%. Similar to other musk deer mitogenomes, a tandem repeat was not found in this region (Kim et al. 2017).

To confirm the phylogenetic position of *M. chrysogaster*, RAxML (Stamatakis 2006), and MrBayes version 3.2.2 (Ronquist et al. 2012) were used on the concatenated datasets of 13 PCGs to reconstruct maximum likelihood (ML) tree and Bayesian inference (BI) tree, by referencing the best-partitioned scheme and optimal model analyzed in Partitionfinder version 1.1.1 (Lanfear et al. 2012). *Hippopotamus amphibius* (GenBank: AP003425) and *Camelus bactrianus* (GenBank: EF507798) were selected as outgroups. The ML tree reflected the same structure as the BI tree. The phylogenetic topology of the families obtained from them was congruent with a previous study in which Moschidae was a sister group to Bovidae (Hassanin and Douzery 2003; Yang et al. 2018). In Moschidae, as the last identified species, *M. cupreus* was at the base and formed a separate clade (Singh et al. 2020).

*M. chrysogaster* is more primitive and is a sister group to *M. moschiferus*, *M. anhuiensis,* and *M. berezovskii* (captive) (Su et al. 1999). From wild populations to those in captivity, more molecular data should be examined on the genetic diversity and geographical distribution of this species to increase musk reserves. The newly sequenced species obtained from Feng County, China, clustered with *M. chrysogaster* (GenBank: KP684123, KC425457) and were closely related to the *M. leucogaster* and *M. berezovskii* (wild) branch, with 94.60% sequence similarity to *M. chrysogaster*_JQ608470 (Miyaluo Nature Reserve, Sichuan, China) and with 99.80% and 99.96% sequence similarities to *M. chrysogaster*_KC425457 (Xinglong Mountain, Gansu, China) and *M. chrysogaster*_KP684123 (Qinghai Lake, Qinghai, China), respectively (Yang et al. 2013; Pan et al. 2015). Extraordinarily, the status of *M. chrysogaster*_JQ608470 was indicated either a misidentification or an unidentified hybrid individual (Figure 1).

**Figure 1.**
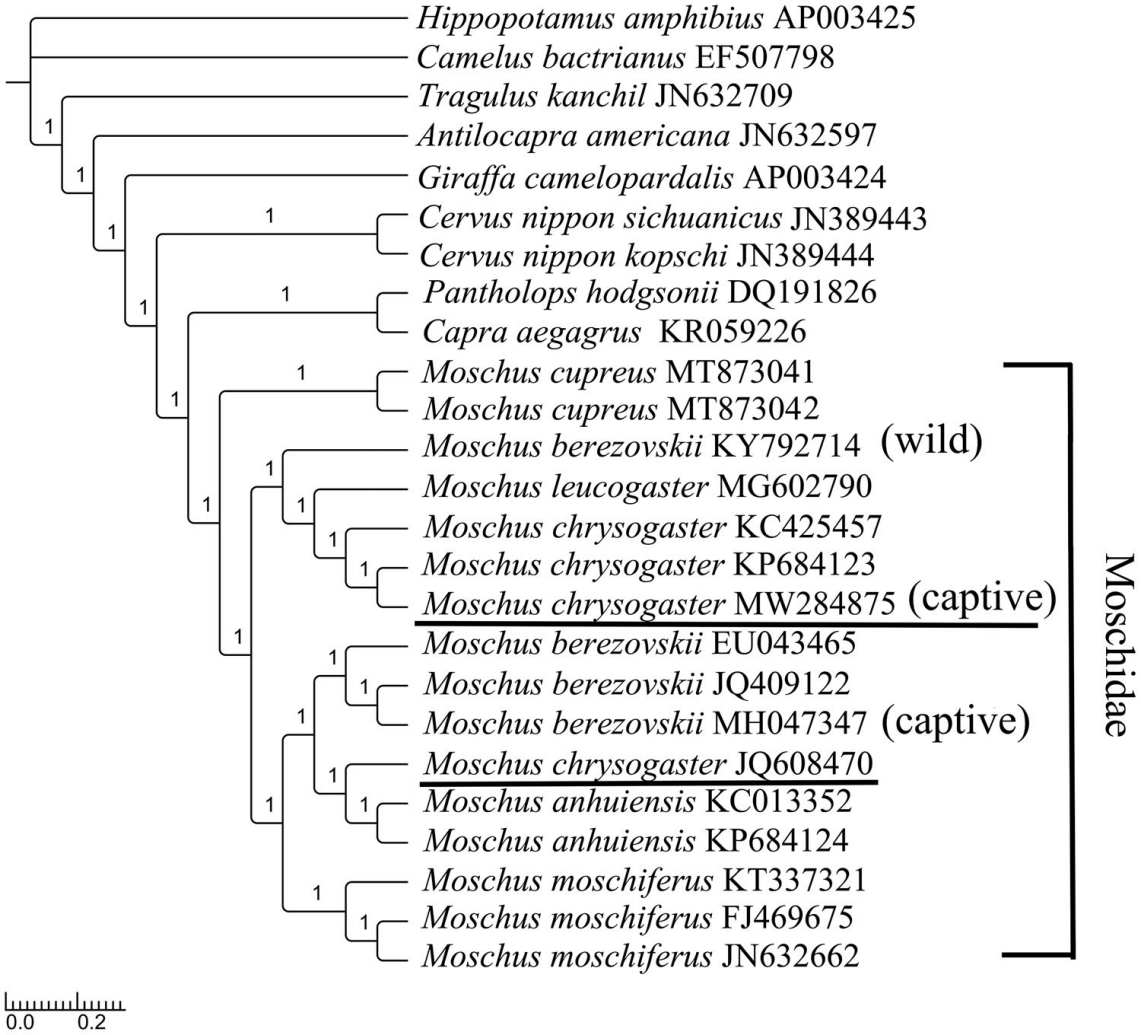
Topology of the Bayesian tree for 25 species based on mitogenome PCG sequences. GenBank accession numbers are indicated following species names (numbers on nodes are bootstrap values).

## Data Availability

The genome sequence data that support the findings of this study are openly available in NCBI GenBank (https://www.ncbi.nlm.nih.gov/) under accession no. MW284875. The associated BioProject, SRA, and Bio-Sample numbers are PRJNA685635, SRR13258650, and SAMN17088691, respectively.
